# MiasDB: A Database of Molecular Interactions Associated with Alternative Splicing of Human Pre-mRNAs

**DOI:** 10.1371/journal.pone.0155443

**Published:** 2016-05-11

**Authors:** Yongqiang Xing, Xiujuan Zhao, Tao Yu, Dong Liang, Jun Li, Guanyun Wei, Guoqing Liu, Xiangjun Cui, Hongyu Zhao, Lu Cai

**Affiliations:** 1 School of Life Science and Technology, Inner Mongolia University of Science and Technology, Baotou, 014010, China; 2 School of Science, Inner Mongolia University of Science and Technology, Baotou, 014010, China; University of Valencia, SPAIN

## Abstract

Alternative splicing (AS) is pervasive in human multi-exon genes and is a major contributor to expansion of the transcriptome and proteome diversity. The accurate recognition of alternative splice sites is regulated by information contained in networks of protein-protein and protein-RNA interactions. However, the mechanisms leading to splice site selection are not fully understood. Although numerous databases have been built to describe AS, molecular interaction databases associated with AS have only recently emerged. In this study, we present a new database, MiasDB, that provides a description of molecular interactions associated with human AS events. This database covers 938 interactions between human splicing factors, RNA elements, transcription factors, kinases and modified histones for 173 human AS events. Every entry includes the interaction partners, interaction type, experimental methods, AS type, tissue specificity or disease-relevant information, a simple description of the functionally tested interaction in the AS event and references. The database can be queried easily using a web server (http://47.88.84.236/Miasdb). We display some interaction figures for several genes. With this database, users can view the regulation network describing AS events for 12 given genes.

## Introduction

Since the discovery that the number of genes in a genome is not linearly correlated with the complexity and functional diversity of an organism, alternative splicing (AS) has increasingly attracted the interest of researchers. AS, which is widespread in the human genome, has been investigated intensively for many genes and according to recent estimates, over 95% of human multi-exon genes undergo this process [1–3]. AS significantly complicates the processing of pre-mRNA. In higher eukaryotes, AS of pre-mRNAs is essential for regulating gene expression, as it alters the function of a gene in different tissues and developmental stages by generating various mRNA isoforms composed of different combinations of exons. Indeed, AS plays an important role in numerous processes, including cell proliferation, apoptosis, development, and differentiation [4–5], and dysregulation of AS leads to a number of human genetic diseases [6–8].

The process of removing intron and joining exons to form mature mRNAs occurs in the nucleus and is accomplished by five small nuclear ribonucleoproteins (U1, U2, U4, U5 and U6 snRNPs) and more than two hundred proteins through the step-by-step assembly of the spliceosome [9]. Recognition of a 5’ splice site involves a base-pairing interaction between the 5’ splice site sequence and the snRNA component of the U1 snRNP. The first step in the recognition of the 3’ splice site is the binding of splicing factor 1 (SF1) to the branch point sequence (BPS). Then, the 65 kDa U2AF subunit binds the polypyrimidine tract (PPT), while the 35 kDa subunit contacts the AG at the end of the intron. Next, the U2 snRNP displaces SF1 and interacts with the BPS through base-pairing. The U4/U6 and U5 snRNPs are then recruited as a preassembled U4/U6.U5 tri-snRNP and, after rearrangement, form the catalytically active complex to perform the chemical reactions of splicing [10]. Although U2-type introns coexist with U12-type introns in most eukaryotes, the latter account for less than 0.5% of all introns in any given genome. U12-type introns are processed by a specific U12-dependent spliceosome, which is similar to, but distinct from, the major U2-dependent spliceosome [11–13]. AS events can be categorized into seven major types: (i) exon skipping; (ii) alternative 3’ splice site; (iii) alternative 5’ splice site; (iv) intron retention; (v) mutually exclusive exon; (vi) alternative first exon; (vii) alternative last exon [14].

The well understood mechanisms of AS regulation involve interactions between splicing factors (SFs) and their target RNA elements [15–17]. Strong splice sites are more efficiently selected than weak, or sub-optimal, splice sites, and alternative exons are frequently associated with the latter. The recognition of weak splice sites depends on the binding of specific *trans*-factors to *cis*-elements of the pre-mRNA. *Trans*-factors include serine-arginine rich (SR) proteins and heterogeneous nuclear ribonucleoproteins (hnRNPs), etc. The *cis*-elements include exonic splicing enhancers (ESEs), intronic splicing enhancers (ISEs), exonic splicing silencers (ESSs) and intronic splicing silencers (ISSs). Unlike enhancers, silencer sequences such as ESSs and ISSs negatively regulate the inclusion of AS exons by interacting with SFs. Additional proteins that do not directly bind RNA, such as transcription factors (TFs), kinases, and histone-modifying enzymes, have also been shown to regulate AS [17, 18–19].

The construction of AS databases is helpful for the identification, classification, functional annotation, and expression profiling of alternative transcripts and for elucidating the regulatory mechanism of AS. Several AS databases have been constructed, and these resources are currently available to the public on the Internet. Most were developed to identify AS events based on either automated large-scale comparisons of expressed sequence tags (ESTs) extracted from publicly available databanks, such as GenBank, EMBL, or DDBJ, or from mining experimental databases. For example, Hollywood [20], ASD [21], ECGENE [22], ASAP [23], PALS db [24], EASED [25], SPLICEINFO [26], Fast DB [27] and HEXEvent [28] were constructed based on ESTs and AsMamDB [29], ASDB [30] and SpliceDB [31] were constructed by searching experimental databases. However, the alignment algorithms are different among these databases due to the differences in primary sequences. Furthermore, most of these AS databases are incomplete because they are largely based on partially and imprecisely sequenced cDNAs (ESTs) or on computationally derived exon information. Other databases that depict AS-induced alterations in protein structures or interactions between RNA and SFs are available. AS-ALPS provides spatial relationships between protein regions altered by AS and the protein’s hydrophobic core and sites of inter-molecular interactions [32].

SpliceAid-F was established by screening the literature; it is currently the only database describing interactions between SFs and their RNA-binding sites [33]. This database includes many artificially mutated RNA elements and does not include any records related to proteins other than those that bind to RNA elements. Furthermore, SpliceAid-F contains only a small number of SFs and focuses on their RNA-binding specificity. Although a large number of molecular interactions associated with AS have been identified through experimental analysis, AS databases do not generally include this information. Thus, it is increasingly important to create comprehensive databases that include the molecular interactions involved in AS regulation.

By manually screening the literature, we retrieved experimentally validated interactions that regulate human AS events and assembled them into an online database called the database of molecular interactions associated with alternative splicing (MiasDB) (http://47.88.84.236/Miasdb). Our database collected 938 human interactions between RNA elements, SFs, TFs, splicing-associated kinases and modified histones for 173 human AS events. Then, the web server for free browsing was built. MiasDB and a number of other available databases on AS complement each other and are indispensable for many computational biologists and molecular biologists. Data-based inferences of the regulation network describing AS events for a given gene are necessary to extrapolate connections between splicing factors and other signal pathways, and as a proof of principle, we built interaction figures for 12 genes based on MiasDB. Overall, MiasDB provides a comprehensive resource of AS interactions in humans, and this database will aid in uncovering the regulatory principles of splicing processes.

## Results and Discussion

### MiasDB data statistics

MiasDB, launched in January 2016, provides AS interaction information for the human genome and includes a total of 938 interactions of AS in humans, of which 29 are specific for the minor splicing pathway (S1 Table). For each interaction, the database provides basic information including interaction partners (defined as interactors on the webpage), interaction type, experimental technology, AS type, tissue specificity and disease-related information, a simple description on the function of the interaction in the AS event, and references (PubMed ID). Hyperlinks to PubMed are also provided. Although some of the experiments were carried out in non-human mammalian cell models, the interactions also occur in humans; these interactions are flagged as human genes in this database.

MiasDB includes 538 experimentally validated interactions regulating specific AS events for 131 genes. The names of the 131 genes, annotated using approved symbols according to the HUGO Gene Nomenclature Committee (HGNC), are shown in Table 1. A user can link to the HGNC by clicking on ‘gene name’. MiasDB also includes 400 interactions for which the gene name has not been determined.

**Table 1:** Genes included in MiasDB.

The 131 human genes
ACTN1; ADCYAP1R1; AICDA; APP; ARAF; ATM; ATP2A1; ATP5C1; BACE1; BCL2L1; BCL2L11; BCLAF1; CAMK2D; CAPZB; CASC4; CASP2; CAV2; CCND1; CD200; CD247; CD44; CD6; CDC42; CDKN1B; CFTR; CLCN1; CLK1; CSK; CTNND1; CTTN; DAB1; DLG4; ELAVL4; ENAH; ENG; EPB41; FAS; FECH; FGFR1; FGFR2; FN1; FOX2/RBM9; GABRG2; GPHN; GRIA2; GRIN1; HIPK3; HIV-1 TAT; HMGCR; HNRNP A1; HRAS; HTR2C; IGF1; IL7R; INSR; IRF3; ITGA2; KCNMA1; LEF1; MADD; MAG; MAP2K7; MAPK9; MAPT; MCL1; MDM2; MEF2; MEF2D; MST1R; MT-CO2; mTOR; MYBPC3; MYC; MYH10; MYOM1; MYPT1; NACA; NCAM; NDUFB11; NEO1; NF1; NOS3; NOVA1; NRXN1; PAX4; PKM; PLP1; POLDIP3; PPARG; PPP1R12A; PRKCB; PS6KB1; PSEN2; PTBP2; PTPRC; RAC1; RBFOX1; REST; RGS4; RNPC3; RUNX1; S100A12; SCN5A; SCN8A; SGCE; SLC2A2; SLC6A11; SLK; SMN1; SMN2; SNAP25; SNRNP48; SNRNP70; SRSF2; SRSF7; SYNE2; THPO; THRA; TJP1; TLE4; TNF; TNNT2; TPM1; TPM2; TRA2B; TRDN; TTN; UFM1; VEGFA

The 938 interactions were classified into two groups: (1) the interactions between RNA binding proteins in which the proteins also called splicing factors and RNA elements (SF-RNA); and (2) the protein-protein interactions (PPIs) in which the proteins may be the RNA binding proteins or other proteins that do not physically interact with the RNA elements. These other proteins may include TFs, kinases and modified histones, etc. There are 525 SF-RNAs interactions in the first group and 413 PPIs in the second group. Increasing evidence suggests that histone modifications play important roles in modulating AS [34–37]. In MiasDB, PPIs include 21 entries describing interactions between splicing factors and histone modifications, including H3K4me3, H3K9me, H3K9me3, H3K36me3, H3K79me, and H3S10P. Furthermore, 909 physical and 29 functional interactions are included in the current database. Overall, the 342 protein factors included in the database have been shown to regulate AS (Table 2). Protein kinases are important regulators of AS, as reflected by the fact that the database includes 75 interactions involved with kinases. The major splicing types involved in these interactions are exon skipping (411), mutually exclusive exons (65), intron retention (22), alternative 5’ splice site (17), alternative 3’ splice site (8), alternative first exons (5) and alternative last exons (11). The interactions in MiasDB are involved in 22 specific tissues and 22 diseases (S2 Table).

**Table 2:** Protein factors included in MiasDB.

The 342 human protein factors
7SK RNP; 9G8/SRSF7; Acinus; AGO; AGO-1; AKT; ASF/SF2/SRSF1; Atx1; BRD2; BRD3; BRD4; BRDT; Brg1; Brm; BS69; c-Myc; CaMKIV; CAPERα; CELF1/CUG-BP1; CELF2/BRUNOL3/ETR3; CELF3; CELF4/BRUNOL4; CELF5; CELF6; Chd1; Clk/Sty; Clk1-AAA; Clns1a; CPSF5; CPSF; CPSF6; CTCF; CTD; CUG-BP; DARPP-32; DAZAP1; DDx 39b; DDX17; DDX1; DDX3x; DDX41; DDX46; DDX50; DDX5; DGKΔ; Dhx15; Dhx30; Dhx9; Dicer; DSK1; Dyrk1A; E2F1; EFTUD2; Elavl1; Elk-1; ERK; ERK1/2; EWS; EWS-FLI1; Ewsr1; FACTp140; FAST K; Fb1; FIR; Forskolin; FOX1/A2BP1; FOX2/RBM9; FOX3; FUBP1; FUSE-BP; Fus; Fyn; GATA1; Gcn5; GLD-1; Gpatch1; GSK-3β; GSK3; H-Ras; H3; H4; HDAC2; HDAC3; HCC1; HDAC6; HDAC; hLuc7A; hLucA; HMGA1a; hnRNP 2H9; hnRNP A/B; hnRNP A0; hnRNP A1; hnRNP A2; hnRNP A2/B1; hnRNP A3; hnRNP C1/C2; hnRNP C; hnRNP D0; hnRNP E2; hnRNP E3; hnRNP F; hnRNP F/H; hnRNP G; hnRNP G-T; hnRNP GL; hnRNP H; hnRNP H1; hn RNP H2; hnRNP I/PTB/PTBP1; hnRNP K; hnRNP LL; hnRNP L; hnRNP M; hnRNP Q1; hnRNP Q; hnRNP U; HP1γ; HP1α; HP1β; HP1; Hspa1a; Hspa1l; Hspa5; Hspa8; Hu; Hub1; HuC; HuR; Igf2bp3; Ilf3; Jmjd6; JNK1; JNK; Khsrp; KSRP; Luc7L1; Luc7L2; Luc7L3; Manumycin A; Matr3; MBNL; MBNL1; MBNL3; mCLK2; MKK3/6; MNK1/2; MRG15; Mtr4; MYC; N-CoR; Ncbp1; Ncl; NKAP; Nono; Nop56; Nop58; NOVA1; NOVA2; Npm1; nPTB/brPTB/PTBP2; NSD3; nSR100/SRRM4; Nxf1; P-TEFb; Ott1; p32; p38α; p38; p47; p68; p72; p100; PABP1; PABP4; Pabpc1; Pabpc2; PARP; Pcbp1; Pcbp2; PDCD7; PI-3; Pin-1; pinin; PKA; PKC; PM/Scl-100/Rrp6; PP1; PP1γ; PP2γ; Ppp1ca; Ppp1r10; Ppp2r1a; PQBP1; Prmt5; Prp22; Prp2; Prp3; Prp31; Prp4K; Prp4; Prp5; Prp6; PRp8; PRPF6; PRPF40A; PSF; PUF60; PurH; QKI-5; QKI-6; Quaking; RAB3A; RAD51; RANBP9; Ras; Raver1; RBM24; RBM25; RBM35a/ESRP1/ISAR1; RBM35b/ESRP2/ISAR2; Rbm38/RNPC1; RBM39; RBM4; RBM; RBM5; RBMY; RNAP II; RNPC3; RNP S1; Rpl7a; Rplp0; Rps2; Rps3a; RRP1B; Rsd1; RSRC1; SAF-B; Safb2; Sam68; SAP155; SC35/SRSF2; SEK-1; Ser2-P RNP II; Setd1b; SF1; SF3a60; SF3b; Sf3b1; SF3b3; SF3b14; SF3b130; SF3b155; SF3B2; SFK; Sfpq; Sgf29; SH3GLB2; Sip1; SIPP1; SLM1; SLM2; Sltm; Slu7; Sm D1; SM; SMAR1; Sm D3; SMN; SND1; SNRNP35; SNRNP48; Snrpd3; Snu66; SON; sp1; SPF45; SPP2; SRA4; SREK1; SRm160; SRm300; SRp20/SRSF3; SRp30c/SRSF9; SRp34; SRp38/SRSF10; SRp40/SRSF5; SRp46/SRSF8; SRp54/SRSF11; SRp55/SRSF6; SRp75/SRSF4; SRPK1; SRRM1; SRrp53; SRrp86/SRrp508; SSA; SSB; SSRP1; SYN1; T-STAR; Taf15; TAP; Tardbp; TBP; TCERG1; TDP-43; Tdrd3; TIA-1/TIAR; TIA; TIP60; Tmpo; TP53BP1; Tra2β; Tra2-β1; TRAP150; TRBP; Trf4-1; U1 snRNP; U11 snRNP; U1-70K; U1C; U2 snRNP; U12 snRNP; U2AF35; U2AF65; U2AF; U2surp; U4 snRNP; U4atac snRNP; U5 snRNP; U6 snRNP; U6atac snRNP; UAP56; Urp; Wbp11; Wdr77; Wnt/β-catenin; YB-1; YT521-B; Zfr; ZNF265; ZNF638

### Access to the database

MiasDB is a comprehensive information resource describing SF-RNA and protein-protein interactions associated with AS. The data in MiasDB are freely accessible through the web interface, which allows users to access and intuitively browse through the information. The search entry allows users to retrieve interaction information using one of three features: the name of a gene with an AS event, the name of SF or RNA elements, or the AS type (see S1 Fig). The output for each selected feature is displayed in a table. Detailed instructions on the operation process of the database can be found at the help entry on the webpage.

### Comparison with other AS databases

MiasDB has features that clearly distinguish it from other AS databases. Most existing AS databases are aimed at collecting AS events but do not provide information regarding the regulatory mechanisms. SpliceAid-F is the only database that shares some features with MiasDB (Table 3) [33]. However, SpliceAid-F only focuses on the RNA-binding specificity of trans-acting SFs and contains 71 human RNA-binding splicing regulatory proteins, whereas MiasDB includes interactions of SF-RNA and PPIs and contains 342 human RNA-binding splicing regulatory proteins. In addition, SpliceAid-F includes information for multiple organisms, including human, mouse, and HIV-1, etc. In total, 655 human RNA-binding sequences and 111 genes are included in SpliceAid-F. These RNA-binding sequences can be divided into 456 natural binding sites and 199 mutant binding sites; deleting some repeated binding sites results in 331 binding sequences consisting of 236 natural binding sites and 95 mutant binding sites. In 2009, Chen and Manley collected binding sequence information for 18 SR proteins, 14 hnRNPs and 17 tissue-specific AS factors [38]. In total, the advantage of MiasDB compared to other databases can be summarized as follows. (1) MiasDB is mainly concerned with human genome AS. (2) MiasDB not only contains interactions between *cis*-acting elements and *trans*-acting factors but also interactions among *trans*-acting factors. (3) MiasDB stores a larger number of AS interactions and genes than other databases.

**Table 3:** Comparison between MiasDB and other AS databases.

	Inclusion of the molecular interactions in AS regulation	Number of genes	Number of proteins	Type of interactions
MiasDB	Yes	111	342	SF-RNA, PPI
SpliceAid-F	Yes	131	71	SF-RNA
AS events databases*	NA	NA	NA	NA

* AS events database denotes databases constructed by comparing the EST content of transcripts. Number of genes describes the genes for which the molecular interactions of AS regulation are identified.

### Applications of the database

**Fig 1:**
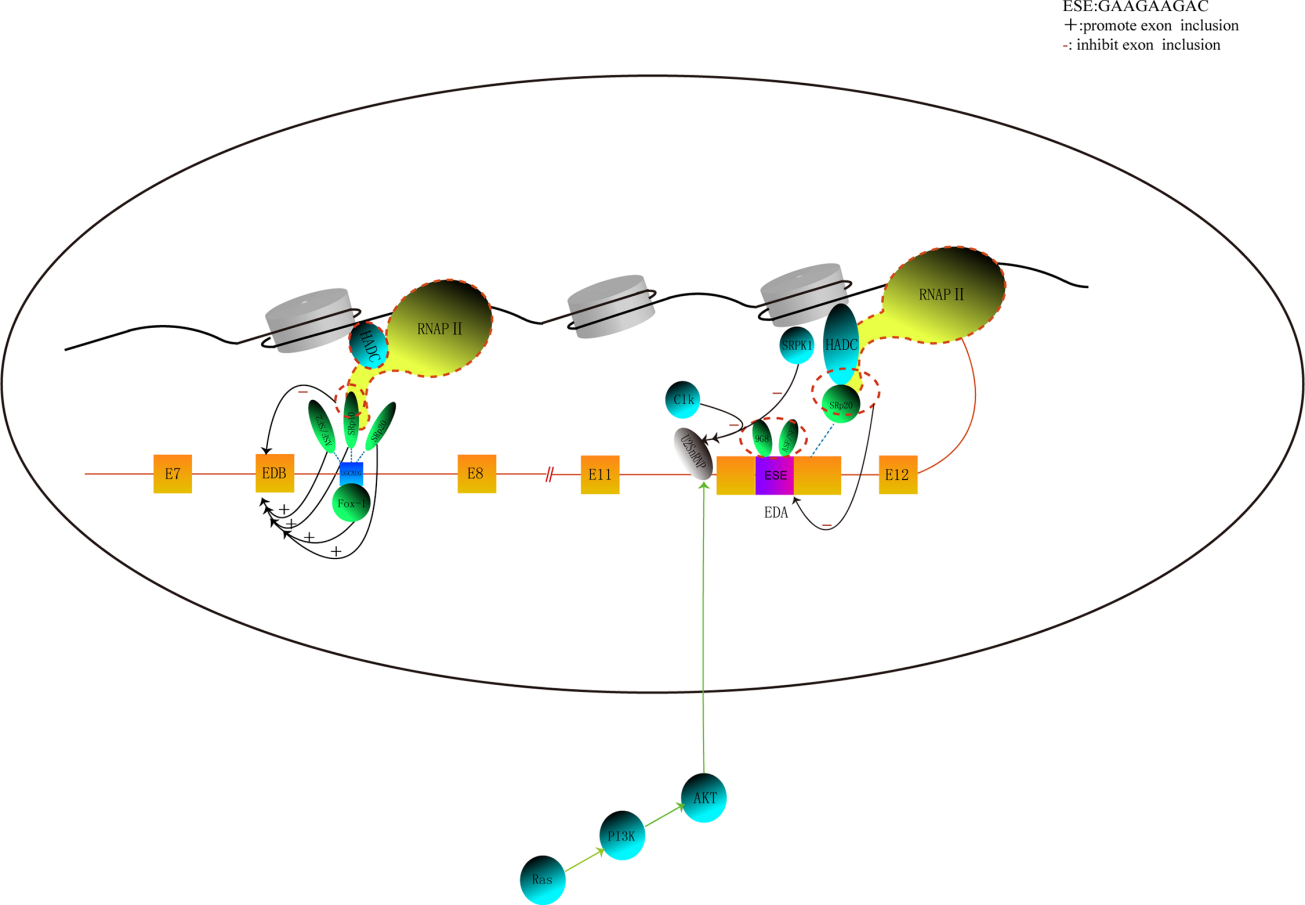
The regulatory network for the alternative splicing of FN1.

MiasDB has many potential applications. One important application is constructing regulatory networks for AS events that involve multiple RNA elements, SFs and other proteins. Examples of networks for specific genes such as *BCL2L1*, *CSK*, *CD44*, *PTPRC*, *CFTR*, *FAS*, *FGFR2*, *FN1*, *INSR*, *NF1*, *SMN2*, and *MAPT* are presented in the current version of MiasDB. A user can observe the regulatory network by searching for the gene name. Here, the regulatory network for *fibronectin 1* (*FN1*) is shown as an example to illustrate the application of MiasDB (see Fig 1). The gene has three alternatively spliced regions: extra domain A (EDA, also known as EDI or EIIIA), extra domain B (EDB, also known as EDII or EIIIB) and type III connecting segment (IIICS or V region). The AS type for EIIIA and EIIIB is the exon skipping. These two exons tend to be excluded in most adult tissues and included during events that involve tissue growth or regeneration, such as embryogenesis and wound healing [39]. The explanation of the AS regulatory network for *FN1* can be downloaded by searching for ‘*FN1*’ in MiasDB.

In Fig 1, the boxes represent exons separated by introns, which are shown as lines. The *cis*-acting elements and *trans*-acting factors regulating FN1 exon selection are indicated. The blue box in the intron downstream of EIIIB denotes an intron splicing enhancer (UGCAUG). The ‘+’ symbol denotes promoting inclusion of the exon; the ‘-’ denotes repressing inclusion of the exon. Direct physical interactions are depicted as a solid line, whereas functional interactions are shown as dotted lines. The black oval denotes the boundary between the nucleus and cytoplasm. Other regulatory networks can be queried by searching for the gene name in MiasDB.

### On-going developments and future directions

Although many AS databases have been developed over the past few years, most were constructed by comparing the EST content of transcripts from the same gene. Databases including information regarding AS regulation remain scarce. In this regard, MiasDB provides a comprehensive database that describes the interactions among RNA, SFs and other protein factors in AS regulation. Accordingly, MiasDB is helpful for constructing AS regulatory networks and provides a guide for experimental investigations of the mechanisms that regulate AS.

MiasDB release 1.0 will serve as a central resource for AS factor interaction. Updates, improvements and further developments will be performed annually. We will continue to update the interaction information related to human AS events, and in the future, we expect to add interaction information for other organisms via carefully curated screenings of the literature. In addition, a linkage between MiasDB and other databases, such as KEGG (Kyoto Encyclopedia of Genes and Genomes), will also be built. The existing network of AS mechanisms and the analytical capabilities of the web interface will be expanded with further novel data-mining and visualization tools. Due to the cotranscriptional nature of splicing, splicing factors and transcription factors can influence each other, thus we will also include information regarding interactions between SFs and TFs. By integrating information on splicing pathways in MiasDB release 1.0 and other related databases, we will also develop theoretical models to infer new nodes and edges in the network.

### Availability

The database is freely accessible through the web server at http://47.88.84.236/Miasdb. Furthermore, all metadata records, statistics and supporting information for MiasDB have also been uploaded to Figshare. The URLs at which data from MiasDB can be accessed in Figshare is ‘https://dx.doi.org/10.6084/m9.figshare.3103057.v1‘.

## Materials and Methods

### Database resources

In MiasDB, all of the interaction information associated with AS was obtained from literature in which the interactions were experimentally validated. We performed searches in PubMed resources by entering the term ‘alternative splicing’. Several thousands of papers published before January 2016 were screened, and 330 publications containing experimentally validated interaction information among RNA, SFs and other protein factors on AS events were used to populate the database.

### Design of the MiasDB interface

The web frontend of MiasDB was created in HTML with PHP language. The database was developed under a relational database framework using MySQL. The interface consists of five different sections (see S2 Fig): a ‘home page’ to introduce the database, ‘Database Statistics’, a ‘search’ entry to query the database and present the results of a query, ‘Help’ to provide instructions on the operation process, and ‘Contact Us’ to show the correspondence information for our group.

## Supporting Information

S1 Fig Search system of MiasDB. (DOC)

S2 Fig Main page of MiasDB. (DOC)

S1 Table Interactions in the minor splicing pathway. (XLSX)

S2 Table Specific tissues and diseases relevant to human AS. (DOC)

## Abbreviations

AS: alternative splicing
SF: splicing factor
MiasDB: the database of molecular interactions associated with alternative splicing
