# Supplementary figures and images for: MiasDB: A Database of Molecular Interactions Associated with Alternative Splicing of Human Pre-mRNAs
Source: PLoS One. 2016 May 11;11(5):e0155443. doi: 10.1371/journal.pone.0155443 (PMC4864242; doi:10.1371/journal.pone.0155443)

**S1 Fig. Search system of MiasDB.**


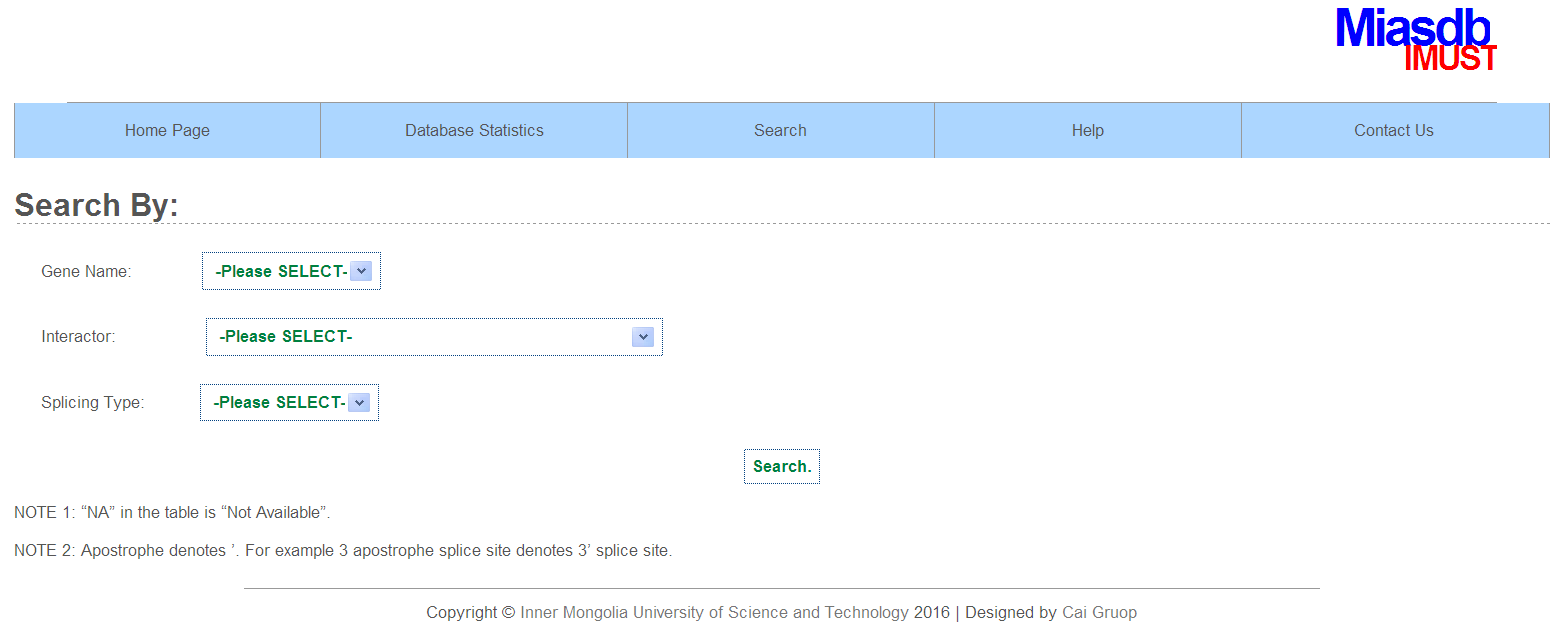

Supplement: S1 Fig — (DOC) [file pone.0155443.s001.doc]

**S2 Figure. Main page of MiasDB.**


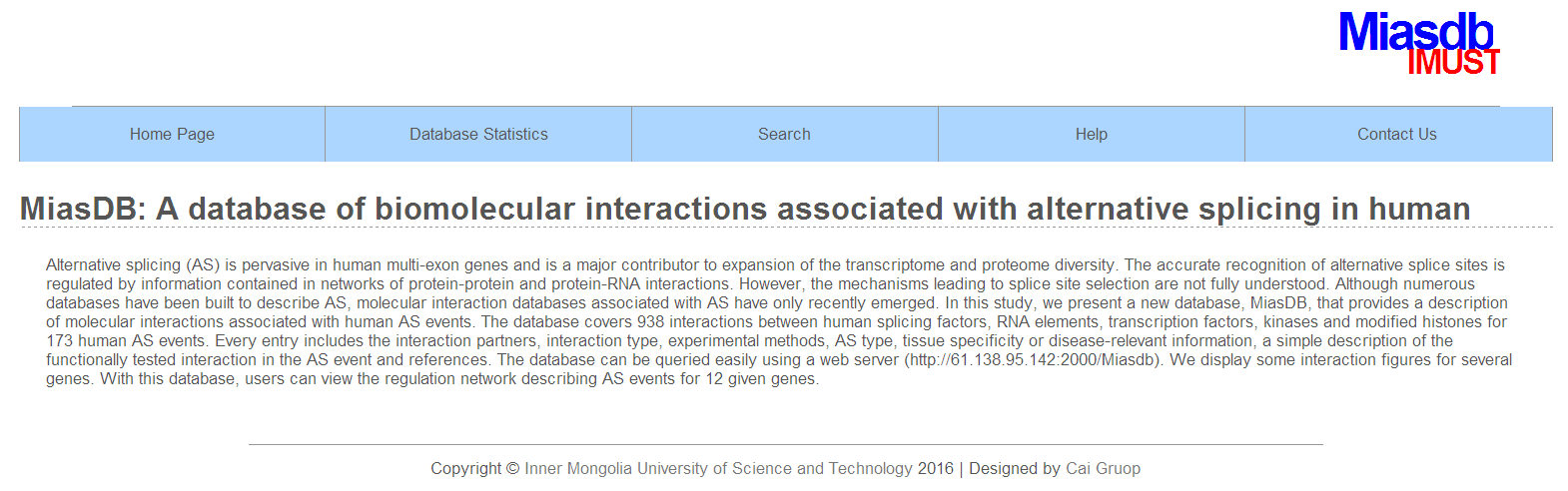

Supplement: S2 Fig — (DOC) [file pone.0155443.s002.doc]
