# Supplementary material for: MiasDB: A Database of Molecular Interactions Associated with Alternative Splicing of Human Pre-mRNAs
Source: PLoS One. 2016 May 11;11(5):e0155443. doi: 10.1371/journal.pone.0155443 (PMC4864242; doi:10.1371/journal.pone.0155443)
Supplement: S2 Table — (DOC) [file pone.0155443.s004.doc]

**S2 Table. Specific tissues and diseases relevant to human AS.**

| Specific tissues | Diseases |
| --- | --- |
| adipose; brain; breast; colon; embryo; epithelium; head; heart; lung; mesenchyme; muscle; glia; myocyte; neck; nervous system; pancreas; prostate; skeleton; spinal cord; testis; vessel; ovary | Alzheimer; autoimmune; breast tumor; cardiomyopathy; colorectal tumor; cystic fibrosis; glioblastoma multiforme; colon cancer; head cancer; demyelinating; neck cancer; lung cancer; myotonic dystrophy type 1; neuroblastoma; non-small cell lung cancer; prostate cancer; small cell lung cancer; spinal muscular atrophy; spinocerebellar ataxia type 1; X-linked mental retardation and neurodegenerative diseases; neural and muscular degenerative diseases; ovarian cancer |
